# Measuring the iceberg: complex lives, invisible metrics, and lived experience in social prescribing

**DOI:** 10.3389/fpubh.2026.1800306

**Published:** 2026-03-25

**Authors:** Robert Dean

**Affiliations:** School of Creative Arts, University of Lincoln, Lincoln, United Kingdom

**Keywords:** creative health interventions, evaluation, qualitative research, relational labour, social prescribing, thematic analysis, wellbeing

## Abstract

**Introduction:**

Social prescribing has emerged as a prominent public health intervention in the UK and internationally, intended to address non-medical determinants of health and reduce pressure on healthcare systems. Despite its rapid expansion, the evidence base underpinning social prescribing remains contested. Dominant evaluation frameworks tend to prioritise quantitative indicators such as referral volumes, attendance rates, changes in standardised wellbeing scores, and short-term service utilisation. While these measures provide policymakers and commissioners with familiar forms of evidence, they often fail to capture the forms of change participants themselves describe as most meaningful.

**Methods:**

This study uses the metaphor of the iceberg as a critical analytic lens to examine what remains invisible within prevailing evaluation regimes. Drawing on forty in-depth qualitative interviews with individuals engaged in social prescribing, the study explores experiences of referral, engagement, perceived impact, and the role of link workers in supporting participation. Thematic analysis focused on how participants interpreted social prescribing within the broader context of their lives and how this shaped engagement and perceived outcomes.

**Results:**

Three interconnected themes emerged. First, engagement frequently occurred against a backdrop of cumulative and overlapping life struggles, including trauma, chronic illness, poverty, social isolation, and experiences of systemic exclusion. Second, participants described profound and often unexpected forms of change that extended beyond standard wellbeing outcomes, including increased feelings of safety, restored trust, renewed purpose, and the prevention of further deterioration or crisis. Third, the relational labour of link workers was central to enabling engagement and sustaining fragile forms of progress over time.

**Discussion:**

By foregrounding lived experience, the findings challenge reductionist models of evidence and suggest the need for a recalibration of how social prescribing is evaluated. The study highlights the importance of recognising stabilisation, prevention, and relational processes as legitimate forms of impact, which are currently underrepresented in the evidence informing public health policy, commissioning, and practice.

## 1 Introduction

Since the NHS prioritised social prescribing as part of its Long-Term Plan in 2019 [NHS (1)], it has become an established public health policy in the UK. It is positioned as a means of reducing pressure on overstretched health services by addressing health and wellbeing needs through non-medical interventions (2). Its rapid expansion within the UK has been accompanied by growing international interest, with social prescribing models now being adapted and implemented across global health systems (3). As a result, social prescribing is increasingly regarded not only as a service-level intervention, but as a scalable policy response to complex, long-term public health challenges.

Despite this momentum, the evidence base underpinning social prescribing remains contested (4, 5). Evaluation frameworks have tended to privilege linear, quantitative indicators such as referral numbers, attendance rates, reductions in GP appointments, and changes in standardised wellbeing scores (6). This pattern is not only common in the UK, but also in international research (7, 8). This is further reinforced in economic evaluations of social prescribing, which focus on short-term cost savings, reductions in healthcare utilisation, and return on investment calculations, often at the expense of longer-term, relational, and cross-sector outcomes that are not easily reduced to monetary value (9). While these measures offer policymakers and commissioner’s familiar data formats, they frequently fail to capture the kinds of change that participants themselves describe as most meaningful, and in some cases as profoundly life changing. Therefore, qualitative research has an important role to play in highlighting the limitations of such approaches to evaluation.

Qualitative studies consistently conclude that social prescribing is best understood not as a discrete intervention with a single measurable outcome, but as a relational, cross sector process in which engagement and perceived benefit depend on person centred tailoring, the quality of the link worker relationship, and the availability and coordination of local community assets (10–13). However, while qualitative studies have been instrumental in foregrounding relational delivery, person centred practice, and cross sector coordination, they have tended to focus on service experiences within relatively bounded frames. Consequently, they capture only partial insights into the cumulative trauma, long-term marginalisation, and systemic exclusion that commonly underly participants’ initial engagement, subsequent improvement, and preventative future proofing that social prescribing provides them with. This contributes to an ongoing disconnect between what is covered within evaluation frameworks and what participants themselves describe as meaningful change. This gap is also evident within policy and practice accounts of social prescribing, which emphasise holistic, person centred responses to complex needs, while offering limited guidance on how such impacts should be evaluated (14). Therefore, addressing this disconnect is not simply a methodological concern, but a policy issue with direct implications for how social prescribing is designed, resourced, and assessed.

To interrogate this, the following paper employs the metaphor of the iceberg as a critical analytic lens. Above the surface lie the visible indicators that dominate policy discourse, such as referral volumes, uptake, attendance, and standardised outcome measures. Beneath the surface lies a much larger body of experience that remains largely invisible within current evaluation models. This includes the weight of participants’ life histories, the rebuilding of trust after repeated systemic disappointment, and the scale of progress made when measured against a cumulative backdrop of inequality and ill health. Used in this way, the iceberg metaphor functions not simply as a heuristic device, but as a critique of prevailing evaluation regimes, revealing the blind spots that shape what is recognised as evidence and what is rendered invisible.

This paper reports findings from forty qualitative interviews conducted with individuals that engaged in social prescribing in Lincolnshire between January 2024 and June 2025. Interviews explored experiences of referral, engagement, perceived impacts, and the role of link workers in supporting participation. Analysis focused on understanding how participants made sense of social prescribing within the wider context of their lives, and what this revealed about the factors that shaped its (in)effectiveness and its impact. Three interconnected themes emerged: (1) the cumulative complexity of participants’ life struggles, (2) the profound impact social prescribing had, and continues to have in their lives, and (3) the central role of link workers in mediating engagement and sustaining fragile forms of progress.

By foregrounding lived experience around these themes, this paper challenges dominant evaluation frameworks that prioritise clear cut, linear metrics instead of attempting to capture complex, relational, and preventative forms of change. As such, this paper offers a reframing of evidence for social prescribing policy and demonstrates that genuinely evidence based public health frameworks must integrate complex qualitative insights as central, rather than peripheral data. Moreover, if the submerged dimensions of social prescribing are not brought into view, policy decisions will continue to be shaped by a small part of the bigger picture, leaving the most meaningful effects of social prescribing hidden beneath the surface.

## 2 Materials and methods

### 2.1 Study design and context

This study adopted a qualitative interview design informed by oral history approaches in order to foreground lived experience. By prioritising participant led narration, the study sought to understand how social prescribing was experienced within the wider contours of participants’ lives, rather than as a discrete or isolated intervention. The research was conducted in Lincolnshire, a predominantly rural and coastal county in England that exhibits many of the structural characteristics associated with rural and coastal health inequalities, including geographical dispersion, pockets of significant socio-economic deprivation, and uneven access to health and social care services (15–19).

### 2.2 Participants and recruitment

Participants were adult service users (aged 18 years and over) who had been referred into Lincolnshire’s social prescribing services. Recruitment was facilitated through link workers who identified potential participants and provided them with information about the study. This allowed individuals to decide whether they were happy to be contacted by the research team, minimised pressure to participate, and helped to maintain the trust participants had in their link worker. Participation was voluntary, and no financial incentives were offered. Individuals who expressed interest were contacted by a member of the research team to discuss the study further and answer any questions. If they were still willing to participate an interview was arranged at a time and location of their choosing. Interviews were conducted according to participant availability and researcher capacity. All interviews were completed before formal the thematic analysis began.

Participants were approached at varying stages of their social prescribing journey. However, none were recruited at the point of initial referral. Most participants were invited towards the end, or just after their formal link worker referral/support period. Link workers were asked periodically (approximately every 4 months over the course of the project) to inform potential service users about the opportunity to participate. In addition, the lead researcher attended several community facing events frequented by current and former social prescribing service users. At these events the lead researcher personally introduced the project and shared his contact details independently of link worker involvement.

Of those informed about the study by link workers, 39 individuals expressed interest in participation, 26 of which responded when the researcher contacted them and were subsequently interviewed. Another 14 participants came forward following direct engagement at community events, resulting in a total sample of 40 participants. Neither the total number of service users approached by link workers, nor the number present at community events attended by the lead researcher was documented. Interviews were conducted independently of anyone involved in social prescribing service delivery.

**Figure 1:**
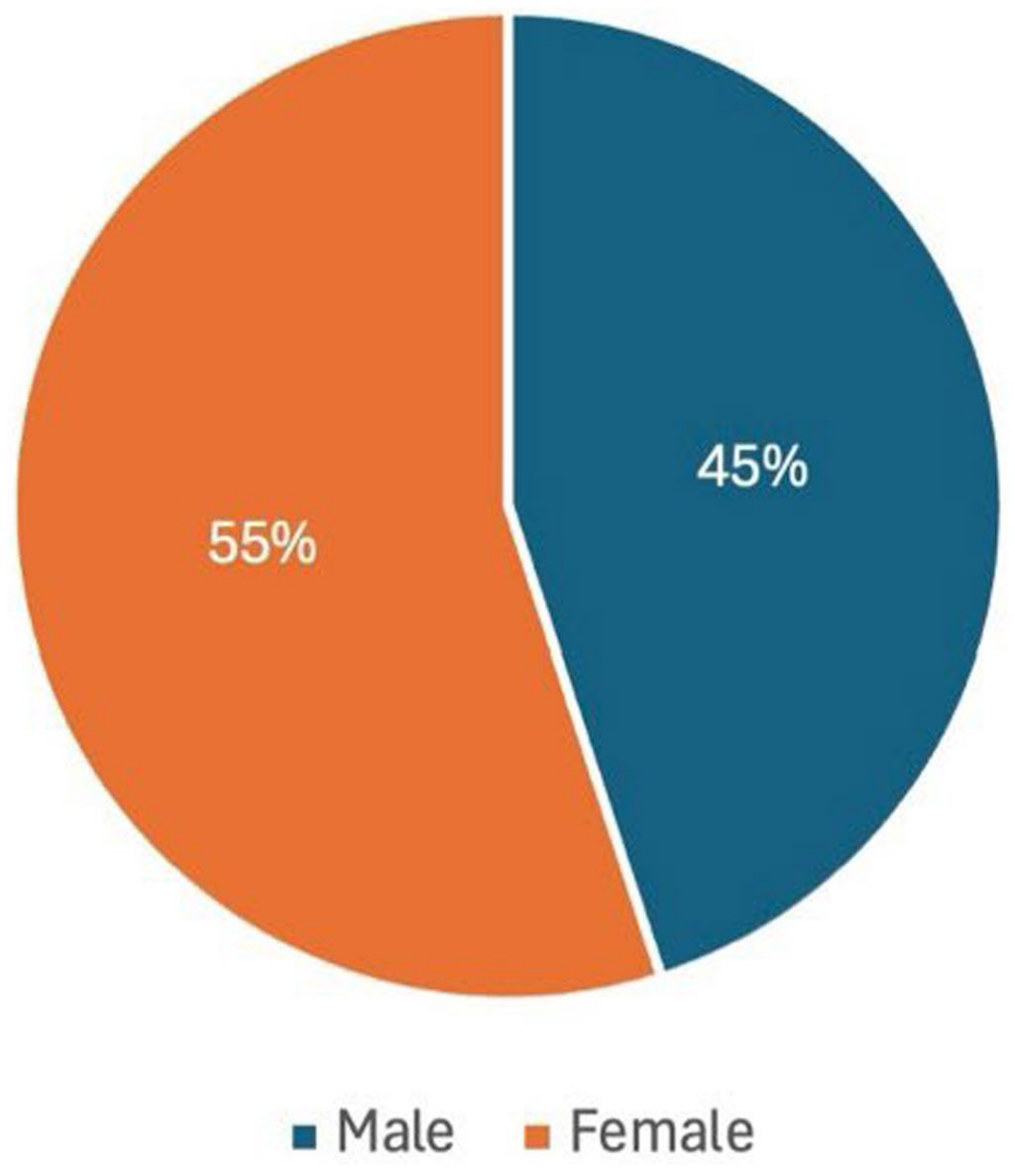
Gender distribution.

**Figure 2:**
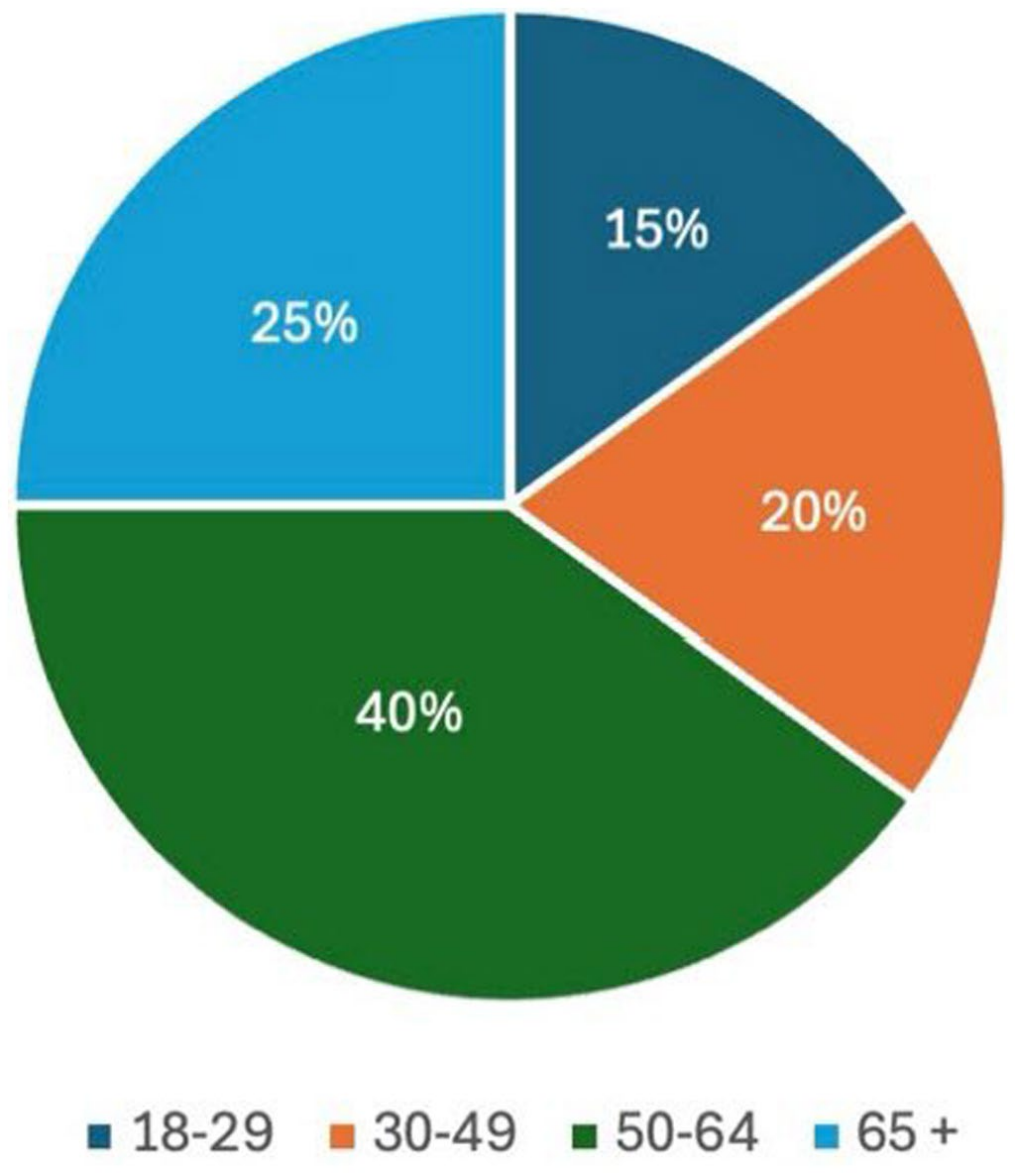
Age distribution.

**Figure 3:**
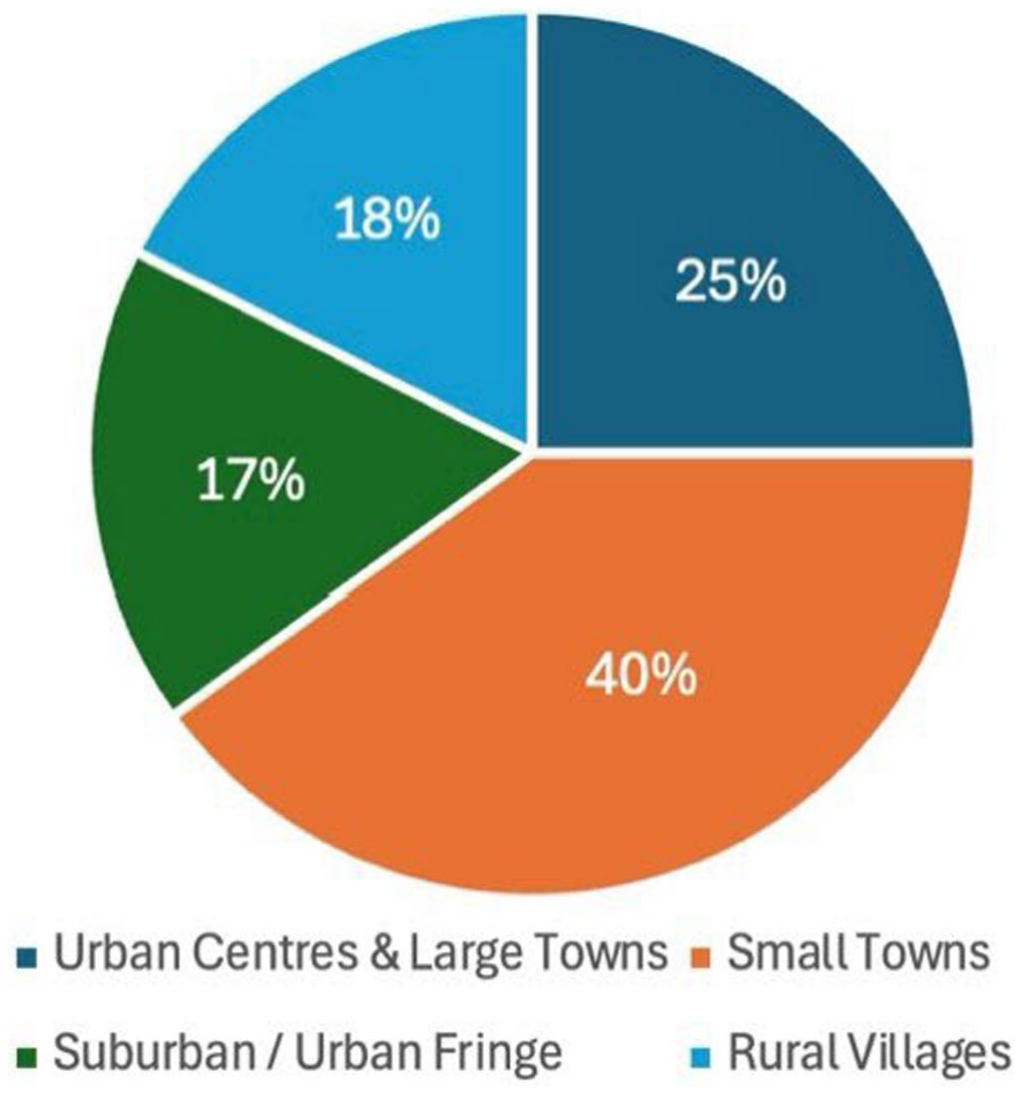
Geographic distribution.

The final sample of 40 participants was not a preset target but reflects a sustained recruitment effort across the 18 months that the project ran. Repeated outreach was required, and not all individuals who initially expressed interest proceeded to interview. The 40 completed interviews therefore represent the cumulative outcome of ongoing recruitment and attrition over time. The overall demographic composition of the sample was not targeted, monitored, or influenced during recruitment. The resulting sample simply reflects those who volunteered to participate. However, as shown in Figures 1–3 (generated by the author), gender, age, and geographic distribution were relatively balanced.

### 2.3 Data collection

All forty interviews were conducted in person by either the principal investigator or the co-investigator (a community connector from a Lincolnshire based charity). Interview locations were determined by participant preference and included community centres, libraries, arts venues, university spaces, and the participant’s own home. In some cases, transport was also provided. Conducting interviews in settings chosen by the participant was intended to support comfort, reduce power imbalances, and foster open, trusting conversations. Interviews lasted between 30 and 70 min and once the participant had given their consent the audio was recorded. Rather than having a rigid question set, a semi-structured interview guide was used as a flexible prompt. As such, the interviews were largely participant led, with most choosing to situate their experiences of social prescribing within broader life histories. These narratives frequently included reflections of childhood experiences, long-term health and social challenges, previous encounters with healthcare, the impact of social prescribing, and the relationship with their link worker. Allowing interviews to develop organically was central to capturing the complexity and meaning of participants’ experiences.

### 2.4 Data analysis and ethics

Audio recordings were transcribed verbatim. An inductive, data-driven, thematic analysis was employed, thereby allowing themes to develop without the use of a pre-existing coding framework (20). Analysis focused primarily on semantic themes grounded in participants’ explicit accounts. These were developed as interconnected, rather than discrete, to reflect the overlapping and cumulative nature of participants’ experiences. A fundamental principle was that participants’ voices should serve as the analytical touchstone. Therefore, over-interpretation was to be avoided, and the ethical responsibility involved in representing lived experience foregrounded (particularly where accounts involved trauma and long-term adversity).

All transcripts were coded in full by the principal investigator. Thematic analysis followed a structured, multi-stage process consistent with Braun and Clarke’s “ongoing” reflexive approach (2006, 82). Following the completion of the interviews, transcripts were written and then read in full to support familiarisation with the data. Initial codes were generated inductively and applied systematically across all transcripts. Codes were then examined for conceptual overlap and grouped into provisional thematic clusters. Through repeated engagement with the data, these clusters were refined, merged, and redefined as patterns became evident across the interviews. Analytic notes were recorded to document interpretive decisions and track the development of themes. The final thematic structure reflects patterns most strongly and consistently represented across participants rather than isolated accounts.

Ethical approval for the study was granted by the University of Lincoln Research Ethics Committee (Reference: LEAS:2023_01_16279). Prior to interview, all participants received a detailed Participant Information Sheet and provided informed consent. Participation was voluntary, and participants were reminded of their right to withdraw at any time, and up to 1 week following the interview. Given the potential for interviews to involve sensitive topics, procedures were put in place to minimise distress. Participants could pause or stop interviews at any point, and information about support services was provided as part of the debrief process. Given the highly sensitive nature of some disclosures (including experiences of violence, abuse, and criminal victimisation), extended verbatim quotations have been selectively limited in sections addressing trauma to reduce risks of identification and to avoid reproducing material that could cause distress or compromise participant confidentiality. Interpretive claims in these sections are grounded in the full dataset but are presented with careful attention to ethical responsibility.

### 2.5 Reflexivity and researcher positionality

Neither the principal investigator nor the co-investigator was employed by, commissioned by, funded by, or involved in the delivery of social prescribing services. No pre-existing relationships existed between the researchers and participants prior to recruitment. The co-investigator’s professional role as a community connector was situated within a separate charity organisation (Every-One) and was not part of social prescribing provision. The co-investigator did not recruit participants and conducted interviews only at the request of the principal investigator. Both researchers have experience conducting qualitative interviews and focus groups within wellbeing and mental health contexts.

The principal investigator has formal postgraduate training in psychological research methods and experience conducting and leading commissioned qualitative research. They also have an established academic interest in social prescribing and teach the subject to undergraduate medical students. While this interest is informed by a belief that social prescribing can be beneficial, this positioning was subject to ongoing reflection throughout the project. Critical, negative, and contradictory accounts were incorporated into the analytic process and informed thematic refinement. Reflexive notes were made whilst coding to document interpretative decisions. Themes evolved through a repeated, cyclical review of the transcripts. The iceberg metaphor and analytic framing were developed after thematic patterns had been identified, rather than being imposed in advance. The reporting of this study was informed by established qualitative research reporting standards to support transparency and methodological clarity.

## 3 Results

Although this study was not designed to produce statistical generalisability, the recruitment of 40 participants has allowed for the reporting of descriptive demographic data and the prevalence of themes within this sample. The figures reported in the Results section are descriptive and were derived from counts across fully coded transcripts following completion of analysis. These figures are included to contextualise patterns within the data rather than to imply generalisability beyond the participants interviewed. Furthermore, as the aim of the study is to bring to the surface forms of experience and change that are often overlooked by standardised evaluation measures, analytic breadth was prioritised over detailed case study analysis. As such, rather than providing exhaustive narrative accounts of individual cases, the analysis seeks to clarify how social prescribing outcomes are experienced across a large and varied group of participants.

### 3.1 Participant characteristics and study context

The study comprised 40 adult participants who engaged with social prescribing services in Lincolnshire between January 2024 and June 2025. 22 participants identified as female and 18 as male, reflecting a slightly higher representation of women within the sample (Figure 1). Participants spanned a wide range of ages. Six were aged 18–29, eight were aged 30–49, sixteen were aged 50–64, and ten participants were aged 65 and over (Figure 2). Participants were geographically dispersed across Lincolnshire. Twenty-five percent (*n* = 10) were drawn from urban centres and large towns, 40% (*n* = 16) from small towns, 17.5% (*n* = 7) from suburban or urban fringe locations, and 17.5% (*n* = 7) from rural villages and small settlements, including coastal hinterland communities (Figure 3).

### 3.2 Service engagement context

The interviewed participants involvement with social prescribing ranged from approximately 6 weeks to 9 months, with a small number engaged for longer periods (up to 16 months) where initial contact attempts were delayed or where engagement unfolded gradually over time. Referral reasons were diverse but most related to mental health difficulties, including depression, anxiety, trauma-related distress, and episodes of crisis. Social isolation and loneliness were also frequent drivers of referral and often linked to bereavement, physical illness, or major life disruptions. Chronic physical health conditions, mobility limitations, neurodiversity, and practical crises such as housing insecurity or financial hardship were also frequently cited. In nearly all cases, referrals occurred at a point when participants were experiencing overlapping and compounding challenges rather than a single issue. The activities and forms of support accessed were also varied. Participants engaged in creative programmes (such as music, art, and crafting activities), physical and nature-based initiatives (such as walking groups, gym membership, and gardening), volunteering and employment pathways, peer support and community groups, and practical assistance with housing, welfare, and benefits. Many participants engaged in multiple forms of support simultaneously.

### 3.3 Routes into social prescribing

Participants described a range of referral pathways into social prescribing, but the most common route was through their GP. Others described referrals initiated within mental health services, while a number entered social prescribing through community routes, including carers’ organisations, veterans’ services, health visitors, and information encountered in waiting rooms. A smaller number described self-referral, typically following encouragement from family members or informal prompts to seek additional support. Across these varied pathways, participants consistently described encountering social prescribing at a moment of heightened difficulty. Entry into the service was commonly situated within periods of crisis or acute distress, including experiences of profound loneliness and isolation, recent bereavement, deteriorating mental health, recovery following hospitalisation or injury, the impact of prolonged COVID-19 shielding, caring responsibilities, or unemployment related stress. For 35% of participants, social prescribing was encountered only after other forms of support had failed. Fourteen of the 40 participants described social prescribing as a last resort, emerging after prolonged engagement with healthcare services that they perceived as ineffective or insufficient. In these accounts, social prescribing was not an early or preventative intervention, but an option introduced only when all other pathways had been exhausted.

### 3.4 Low expectations and scepticism at entry

Across these accounts, a consistent pattern emerged. Social prescribing was introduced to the participant at a point of accumulated frustration, rather than optimism. Participants described having already invested considerable time and emotional energy in engaging with services that did not meet their needs. This had led to them having low expectations of further support and although they agreed to social prescribing, they did so with scepticism. Moreover, many recalled having little or no understanding of what social prescribing involved at the point of referral. Their puzzlement at the idea of being “prescribed” non-clinical activities was often citied. As one participant explained, “I didn’t really have any idea what it was […] I found it a bit weird at first”. Several participants recalled reacting negatively when social prescribing was first mentioned, expressing doubts about whether it would be helpful or relevant to their situation. One participant described being “a bit sceptical” and thinking “oh, God” when the option was raised, expressing frustration at the prospect of once again having to recount their experiences “like a broken record.” For others, scepticism stemmed from a history of unmet promises, as one participant explained, “I’ve tried things in the past that I was told would work […] and they hadn’t, so why should I expect this one to?” Low expectations were also shaped by participants’ assessments of their own capacity to engage. Some expressed doubt about whether they would be able to take part due to anxiety, low confidence, or previous negative experiences of group activities, captured in responses such as, “nobody gets me. There’s no point. It’s a waste of time.”

This starting point provides a useful context from which to consider the participants comments on their initial contact with a link worker following a referral, and the fragile starting point from which their involvement developed. Most participants recalled receiving a phone call or text message from their link worker, followed by an in-person meeting. This early interaction was frequently remembered in detail and was associated with feelings of being noticed, taken seriously, and personally supported. Participants described these early contacts as markedly different from the brief and impersonal appointments they associated with the health service. This contrast was captured by one participant, who reflected, “I felt like I wasn’t a waste of his time […] I felt like I was actually being listened to.”

Many participants also spoke of their link worker as not simply providing information or signposting, but actively accompanying them to activities. Participants repeatedly described the act of being accompanied as the decisive factor that prompted them to actively engage with what social prescribing had to offer. For several, the main challenge was not the activity itself, but the act of arriving, entering a space, and meeting new people. The presence of a link worker significantly reduced these fears. One participant, who had been considering attending a community centre for “about a year and a half” but “just couldn’t do it,” described how attending alongside their link worker made engagement possible, explaining that having them “sitting in eyesight, so I knew she was there” enabled them to remain in the space.

However, participants also described initial barriers to engagement. These included delays between referral and first contact, receiving multiple or confusing communications, transport difficulties (particularly in rural areas) and accessibility challenges linked to mobility, sensory needs, or neurodiversity. In some cases, prolonged delays between referral and contact undermined their opinion of social prescribing, with participants waiting so long to be contacted, they had forgotten about the referral altogether. As one participant explained, “[I’d] completely forgotten that I was even on the waiting list”. Several participants described being confused by the initial contact, as they did not know who was calling them or what service they were being referred to. One participant recalled that, “I had so many people calling me, I didn’t even know who I was talking to”. Despite this, where engagement with social prescribing did occur, participants consistently linked it back to a specific moment of personal contact with their link worker.

### 3.5 Accumulated life struggles and compounding adversity

Participants repeatedly emphasised the length of time they had spent coping with adversity. While individual experiences differed in detail, the cumulative nature of trauma, hardship, and long-term marginalisation was consistently represented across participants, irrespective of demographic variation. The language used to describe their lives often highlighted endurance, repetition, and persistence, with metaphors such as “a long, hard road” and reflections that life had been “a long […] hell of a journey.” Participants also described depression and distress developing gradually over time rather than in isolated episodes. Several spoke of difficulties that were “growing, growing, growing,” alongside cascading pressures in which “one thing [came] after the other.” One participant described successive setbacks as “another failure to build on to all the others,” while another described how “five different things happened” within a short timeframe. This type of experience was reflected in many other accounts in which participants recalled periods of simultaneous, compounding difficulties.

These cumulative trajectories of distress were the backdrop against which most participants began their social prescribing journey. Participants frequently described profound and often overlapping life challenges. Rather than presenting with single or isolated difficulties, their accounts revealed layers of adversity. Histories of trauma were prominent. Participants described experiences of domestic violence, childhood abuse, sexual violence, and exposure to extreme events during early life. For some, these experiences continued to influence mental health, relationships, and capacity for social engagement in adulthood. Trauma was rarely described as a discrete past event. Instead, it was woven into narratives of ongoing distress and vulnerability.

Bereavement and grief featured prominently in participants’ accounts. Several described experiencing multiple losses within relatively short periods of time. One participant shared that they had “a lot of bereavement in a very short space of time,” while another had “just lost both my parents in the space of three months.” Others spoke of their emotional state following the loss of a loved one: “My husband passed away […] I still get upset, I can’t help it”; “my mother passed away […] I was just crying.” For several participants, bereavement was closely linked to acute psychological distress. One recalled feeling “frantic” and “suicidal,” while another shared that they had “only ever wanted to commit suicide once,” and that it followed a bereavement. Another described grief as an embodied experience: “When you’ve lost somebody […] you’re like a fish out of water. You’re just numb.” Participants frequently spoke of grief as ongoing. “My husband died and I haven’t been right since then,” one explained. Another reflected that even speaking about the loss remained difficult: “If I start talking about him, I’m just gonna well up and start crying.” These accounts suggest that bereavement functioned not as a discrete life event but as a destabilising rupture that reshaped participants’ sense of stability and self. In many cases, this occurred alongside other pressures such as declining health, financial insecurity, or caring responsibilities. Social prescribing was therefore often encountered at a point of emotional exhaustion and heightened vulnerability.

Severe mental health difficulties were common across the sample. Participants described depression, anxiety, post-traumatic stress, and periods of acute crisis, including suicidal ideation. These experiences were frequently linked to feelings of hopelessness, exhaustion, and withdrawal from social life. Several had experienced repeated hospitalisation or psychiatric intervention. As one participant stated, “I’ve been in psychiatrics three times now”, another recalled being “put in a psychiatric unit for three months.” Panic attacks and acute anxiety were frequently described: “I had a panic attack”; “I start with panic attacks, and I need to leave”; “I must have had a panic attack […] because the next thing I knew was our doctor was there”; “I […] got a panic attack from going outside.” Others described long-term social withdrawal: “I spent nearly a decade in the house, not getting out”; “I hadn’t left the house since COVID”; “I lost all contact with everyone.” Others spoke of profound exhaustion: “I was constantly tired”; “I wouldn’t get out of bed until about 1:00 in the afternoon”; “I was completely alone.” These accounts indicate that participants were not entering social prescribing from positions of mild distress, but from a place of entrenched psychological difficulty and sustained social disconnection.

Chronic physical illness, pain, and disability compounded these challenges. Participants described living with long-term conditions that limited mobility, independence, and participation in everyday activities. One participant explained, “some days I can’t even get out of bed […] I’m in terrible pain,” while another stated simply, “I can’t walk at speeds. Got arthritis.” Others described restricted mobility in stark terms: “20 metres max [I] can’t walk around a supermarket anymore”; “any distance standing up [I] need a wheelchair.” For some, sudden injuries or neurological conditions marked sudden shifts in their lives. Participants described waking from surgery paralysed, experiencing spinal strokes, or requiring emergency brain operations. Another described being hospitalised for months following a stroke and moving directly into residential care. These physical struggles were often accompanied by a loss of independence. Participants spoke of losing driving licences, employment, homes, and relationships: “Lost my driving licence, lost my car […] lost access to my son. Lost my home.” Others described being forced to leave long held occupations due to injury or disability. Such accounts indicate that many participants encountered social prescribing not only in the context of psychological distress, but amid substantial physical limitation and enforced dependency.

Neurodiversity emerged as another common factor that shaped participants’ experiences. Several described late diagnoses of autism, ADHD, or learning differences and reflected upon how undiagnosed neurodiversity had contributed to lifelong struggles. These included, education, employment, and social interaction, as well as ongoing challenges navigating services and group-based activities. As one participant reflected, “someone with social anxiety, ADHD, autism, don’t navigate social situations very well.” Another participant linked their difficulties with social environments and prolonged isolation to late diagnosis and described spending “nearly a decade […] in the house, not getting out, out of touch with everybody, everything”.

Isolation and withdrawal were recurrent themes across participants’ narratives. Many described long periods of social disconnection, including agoraphobia, fear of public spaces, and lives confined to the home. One described being “stuck in the flat, […] stuck in […] the four walls.” Another stated that they “couldn’t even go across to the shops. My confidence was lower than the carpet […] I couldn’t cope with anything.” Participants spoke of lives that had narrowed significantly, with daily routines confined to the home and minimal contact with others. In these circumstances, the prospect of engaging in any form of social or community activity was often described as overwhelming (rather than simply challenging). As one participant explained, “I wouldn’t have even been able to get out of the front door […] I would have maybe got to the door and then just fallen in a heap and cried […] I was petrified.” Material hardship further intensified these experiences. Participants described poverty, benefit dependence, housing insecurity, and, in some cases, homelessness. Financial strain also limited access to transport, activities, and supportive environments.

Twenty-nine of the forty participants referred explicitly to childhood when explaining their present circumstances. This occurred across all age ranges, and these reflections were offered spontaneously as part of participants’ own narrative accounts rather than in response to direct questioning. For many participants, childhood was associated with experiences of trauma and abuse. Participants described exposure to domestic violence, emotional and physical abuse, sexual violence, bullying, and bereavement. These experiences were often recalled with clarity and participants explicitly linked them to long-term impacts on mental health, confidence, and relationships. One participant reflected on years of being told they were “useless” and “a failure” during childhood, while another described witnessing extreme violence at a young age and experiencing persistent bullying throughout school.

Childhood instability and material hardship also featured prominently. Participants described early experiences of housing insecurity, family breakdown, or periods of sofa-surfing during adolescence. For some, these experiences disrupted education and broke support networks. For others, these histories were understood not as isolated events, but as prolonged traumatic experiences unfolding over decades. One participant reflected on this describing how a later therapeutic assessment helped them recognise the cumulative impact of “at least 50 years of mental abuse,” before adding that they had never, “had any real support through my life […] I’ve literally had to deal with it all on my own.” A smaller number of participants referred to childhood experiences that were not overtly traumatic but nonetheless shaped identity, behaviour, and expectations. These included highly restrictive upbringings, early responsibilities, or learned patterns of emotional suppression. Participants reflected on how these early environments influenced their adult relationships, their sense of agency, and their willingness to seek help.

While the majority of childhood references involved adversity, some participants also recalled positive formative experiences, particularly in relation to creative or musical activities encountered early in life. These memories were often invoked when explaining why certain social prescribing activities felt meaningful or familiar. One participant recalled, “I picked up a pencil when I was like four and just never stopped.” Another recalled a school project, “I made a towelling dressing gown at school. That was the last thing I made […].” Others shared musical experiences from their childhood. One participant explained that they had received violin training at school and joined an orchestra, while another had “been playing since I was about 16” and still owned “the same guitar,” adding, “I’ll never sell it […] It’s part of my soul.” For some, music had been central to their identity in adolescence or early adulthood. Participants described teenage drum kits, involvement in performing arts, or playing in bands. Even where formal training had not occurred, participants described longstanding attachments to music: “I’ve just got a big love of music.” These recollections suggest that engagement with creative social prescribing activities was not always entirely new. Instead, for some participants, such activities reactivated earlier interests that had been interrupted by illness, trauma, or life disruption. This may help explain why particular forms of creative engagement were experienced as especially resonant or restorative.

### 3.6 Social prescribing as preventive support

Participants frequently described social prescribing as having a preventive role, intervening at moments of acute vulnerability and averting further deterioration, crisis, or harm. In these instances, rather than describing support in terms of improvement, participants reflected on how social prescribing had prevented outcomes they perceived as imminent and inventible.

While these accounts are analytically significant, the study does not claim to establish causal attribution in a formal or clinical sense. However, they do demonstrate the participants’ own interpretations of the impact social prescribing had on their lives. One participant stated, “if it wasn’t for social prescribing, I wouldn’t be here,” while another reflected that “without it I don’t think I’d be here […] I’d reached rock bottom.” Multiple participants described periods of suicidal ideation or attempts and directly linked social prescribing to preventing that outcome. Some recalled being close to self-harm or death before support began, describing social prescribing as arriving at a moment when they were almost unable to continue. Others reflected on histories of repeated psychiatric admissions and/or substance misuse and described social prescribing as preventing further escalation. In these narratives, prevention was not abstract but concrete and grounded in participants’ perceptions of having narrowly avoided severe harm. One participant described being “completely alone” prior to engagement and attributed their recovery to the social prescribing activity having “brought [them] back out […] to [themselves].” Another stated that they had been “given a second life.” Many participants described sustained and far reaching transformations, these included dramatic increases in confidence, long-term recovery from substance use, and renewed capacity to engage socially. Participants described social prescribing as enabling the rebuilding of lives that had been severely disrupted by illness, trauma, or exclusion. Accounts included restoring routines, re-entering public spaces, taking on voluntary or caring roles, and a new sense of purpose.

### 3.7 Beyond wellbeing: profound and unexpected change

While the participants accounts do not necessarily establish causal attribution, they do demonstrate how participants themselves understood social prescribing as intervening at critical point in their lives. A renewed sense of safety was frequently emphasised, particularly among participants who had previously felt confined to their homes or unable to trust others. Participants described feeling safe enough to leave the house, attend activities, or engage with new people, sometimes for the first time in many years. Participants also described developing a sense of belonging that extended beyond reduced loneliness. Rather than simply feeling less alone, they spoke of being recognised, known by name, and part of local social networks. One participant spoke of knowing their neighbours, “every one of them […] all by first name.” Others described relational familiarity developing over time: “they sort of got to know that I was there and they come and spoke to me”; “I’ve become good friends with them.” Gains in confidence were frequently described with a sense of surprise. Participants spoke of “starting to grow into it,” feeling “comfortable […] to socialise,” or taking on new responsibilities such as volunteering or chairing meetings: “I’m also volunteering now on a Tuesday”; “I chair the meeting on my own.” Physical achievements were similarly framed in terms of disbelief: “I can’t believe I can do that. Can’t believe I can bench press.” Another described independently driving to a new location as “quite incredible.” Creative activities also prompted moments of affirmation. One participant reflected, “you don’t actually realise how good you are.” Another, after listening back to a recording of their singing, described being surprised by their own voice: “It just blew me away […] Really powerful when I listened to it. I said, I didn’t sing that.” Others spoke of rediscovering forgotten skills. Following a music lesson, one participant explained that the activity had “made me realise that I knew a lot more than what I thought I did.”

These changes were frequently accompanied by shifts in how participants perceived themselves. They described feeling more able to advocate for themselves, make decisions, and exert control over aspects of their lives, particularly housing, finances, or healthcare. For some, engagement with social prescribing led to profound reorientations of their identity. This included reframing long standing self-doubt and reconnecting with creative or spiritual expression. For one participant, this was expressed as a renewed sense of self-value, “I feel like I’ve got a life now. Feel like it’s worth living.” Others described developing greater self-belief, “I’m willing to try […] I just need people who give me that chance.” Another reflected on how they were now pushing themselves “out of that safety zone.” For some the shift was articulated as reclaiming control: “I proved them all wrong.” Others framed the change more quietly but no less profoundly, moving from suicidal ideation to renewed attachment to life: “I’ve got too much to live for.” Overall, for the participants interviewed, social prescribing reshaped how they inhabited the world and understood themselves within it. The effects were relational, embodied, and existential in nature, extending beyond symptom reduction or improvements captured through standardised measures, or Likert scale wellbeing ratings.

Identity reorientation appeared to emerge not from singular events but from incremental shifts in participation, recognition, and belonging. These shifts accumulated over time, often through repeated encounters characterised by continuity and relational trust, rather than through any single prescribed activity. The mechanism of change therefore appears less tied to specific interventions and more to sustained relational engagement within a supportive social context. This has implications for how social prescribing is evaluated. If change is cumulative, relational, and unfolding over time, it may not be adequately captured by models that prioritise discrete outcome measures or short-term improvements. Instead, these findings suggest the need for evaluation approaches that capture shifts in identity, agency, and social positioning over time.

### 3.8 Participants’ experiences of the link worker support

Participants used rich and emotionally resonant language to describe their link workers, consistently emphasising relational qualities over professional or procedural attributes. Rather than referring to link workers in technical or clinical terms, participants described them using language associated with warmth, approachability, and human connection. Link workers were characterised as “lovely,” “calm,” “bubbly,” “chatty,” and “full of life”. Participants explicitly stated that they could trust their link worker and felt safe in their presence. For some, this trust developed into relationships described in friend-like or familial terms. Participants spoke of their interactions with link workers as feeling akin to “catching up with a friend”, or that they had “become a part of our family.” A number of participants used nurturing or explicitly supportive metaphors to describe their link workers, referring to them as “maternal,” “an angel,” or someone who “held my hand” during difficult periods. Participants also frequently cited compassion and understanding, describing link workers as “human being[s] with human feelings of […] compassion” and as people who genuinely cared.

All but one participant described their link worker as being fundamentally different from other healthcare professionals they had encountered. Thirty-nine of the forty participants explicitly contrasted their experiences of link workers with those of clinicians, therapists, and other health service staff. Across these accounts, the emotional and relational dimensions of link worker support were foregrounded far more strongly than procedural or organisational aspects of the role. Participants’ language emphasised connection, safety, patience, and humanity, suggesting that the perceived effectiveness of link worker support was inseparable from the quality of the relationship itself.

Participants’ characterisation of link workers as person-centred rather than procedural was closely tied to how support was experienced in practice. Rather than being guided through standardised pathways or assessment tools, participants described support that was tailored, paced, and responsive to the individual’s readiness. The ability to “start at the bottom and ease [their] way in” was seen as particularly valuable. Indeed, several participants emphasised that they were not pressured to act immediately, but were supported gradually, with link workers returning multiple times before participants felt able to engage in activities outside their home. Another important distinction concerned accompaniment and continuity. Participants contrasted link workers’ ongoing involvement with the one-off or time limited nature of other appointments. Link workers were described as visiting participants at home, checking in regularly, and remaining present over time.

Participants also foregrounded the practical, action focused, nature of link workers’ support. Rather than offering abstract advice or signposting alone, link workers were described as actively helping to make phone calls, arranging contacts, attending first sessions, and accompanying participants to activities. Phrases such as “leave it with me” or “I’ll get back to you” were recalled as reassuring, as participants experienced these assurances being acted upon rather than being empty promises. Several participants highlighted what they perceived as link workers going “above and beyond” formal role expectations. Participants also contrasted the practical actions of link workers with what they described as impersonal or procedural responses from other services. Several recalled previous experiences of being given leaflets, referrals, or advice without any follow up. These accounts described link workers as committed beyond standard working hours or administrative boundaries, which was perceived as evidence that they were being taken seriously and not treated as cases to be processed.

Across these accounts, persistence was consistently highlighted as the difference between engagement and disengagement. Rather than withdrawing after an initial refusal, link workers were described as leaving the door open in ways that felt non pressuring and respectful. This approach was described as being pivotal in allowing participants to engage when they felt ready, and removing the fear of being judged or written off. It was also noted that encouragement was delivered gently rather than coercively, with one participant making sure they clarified that the persistence of their link worker, was not experienced “in a bullying way”. This gentle persistence was important for participants who felt vulnerable or anxious, as it allowed them to engage at their own pace in the knowledge that support remained available. Several participants described initially declining social prescribing or avoiding contact altogether, due to uncertainty, anxiety, or mistrust. In these cases, link workers were described as continuing to follow up rather than disengaging after an initial refusal. Participants recalled being contacted again weeks later, receiving multiple phone calls before meetings were arranged, or being offered the opportunity to reconnect at a later point. For some, this continued contact was credited as the reason engagement eventually occurred, with participants reflecting that without this persistence they would not have engaged with interventions that have come to be hugely important to them.

While most participants described link worker relationships in strongly positive terms, 12 reported episodes of disruption, limitation, or less effective engagement. Poor relational fit itself was rare. Where it occurred, it tended to involve mismatches in activity type rather than a breakdown in interpersonal rapport. Participants described feeling disengaged from certain offers: “arts and crafts [are] not for me”; “I wasn’t so much interested in the arts and crafts”; “this isn’t for me. I’m just wasting time sitting here”; “Some [activities] sounded […] boring”. In two instances, communication style was also noted. Reflecting on a link worker’s approach, one participant commented that “you can’t push when someone is […] clearly telling you they are unwell,” while another described being given a “bag of leaflets” for activities they could not access.

More commonly, however, limitations were structural rather than relational. Practical barriers were often raised. This included difficulties with transport, timing, and medication schedules that restricted engagement: “I didn’t want to travel all the way […] on my own”; “the things that were interesting to me, I couldn’t get to”; “it’s at night and I take my medication at 7 o’clock”; “Suddenly my back went.” Short programmes were repeatedly described as inadequate for ongoing needs: “They gave me eight lessons […] and it stopped”; “It’s a shame it all comes to an end […] where do I go from here?” One participant described being signed off as emotionally destabilising: “When we had to sign me off, that was hard […] I’ve gradually started going downhill.” The loss of a trusted link worker emerged as a particularly significant rupture. Participants described abrupt endings in relational terms: “I miss her. I do miss her”; “I actually got emotional […] It is tough because you do lose your handler”; “she sees you for about six weeks and then she finishes with you and then you’re on your own again.” From these reports it is clear that where relational continuity was broken, the very mechanism participants identified as transformative became a destabilising influence. However, these accounts do not contradict the central findings of this study. Instead, they suggest that relational labour is most effective when continuity is maintained, programme duration aligns with need, and structural barriers do not exceed participants’ readiness or capacity to engage. Where these conditions are absent, even well-matched relational support may be insufficient to sustain engagement.

## 4 Findings

### 4.1 Rethinking impact in social prescribing evaluation

The findings presented in this paper challenge the commonly used approaches to evaluating social prescribing, not simply on methodological grounds, but on the basis of what such approaches are able to recognise and measure. Indeed, in some cases, they may actively misread impact, for example when increased engagement with health or social care services reflects growing confidence, unmet need being addressed, or improved self-advocacy, rather than failure or dependency. This is not to suggest that standardised measures are invalid or without value. However, when used in isolation they provide a partial and often misleading account of the actual impact of a patient’s social prescribing journey if their evaluative gaze is misaligned with lived experience.

It is this mismatch that informs the iceberg metaphor in the title of this paper, which is used as an analytic critique of prevailing evaluation regimes. Above the surface sit the indicators that dominate policy and commissioning discourse, referral volumes, attendance rates, standardised wellbeing scores, and cost-offset calculations. These evaluative measures are not tailored to fit the treatment in question, they are the traditional methods; tested in non-social prescribing contexts, established in academia, and accepted by funders and policy makers. They are methodologically safe, objectively clean, and efficient to deploy. However, beneath the surface lies a far larger and heavier body of experience: cumulative trauma, repeated systemic failure, fragile trust, and a scale of change represented by survival, stabilisation, and renewed capacity in lives shaped by long-term marginalisation. The problem, therefore, is not simply that some impacts are difficult to measure, but the way current frameworks recognise and represent improvement. Data showing that on average a group of participants response to the question “how satisfied are you with your life” moved 6 points up a Likert scale over a 12-week course of social prescribing, does not capture the ways in which the intervention prevented further deterioration, stabilised lives marked by cumulative adversity, and created the conditions necessary for the gradual rebuilding of a life that felt worth living. Such forms of impact, often relational, preventative, and context dependent, remain largely invisible within many evaluation models. Yet, as demonstrated by the participants’ accounts in this study, these experiences are the rule rather than the exception to it. As evidenced in the demographic data set out at the start of this paper, the gender balance of the sample was almost equal (Figure 1), the age range was diverse (Figure 2), as were the locations participants lived in (Figure 3). However, the themes applied across the dataset and the experiences they described all overlap. So, according to the data, while on a demographic level a 18 yr. old, female living in an urban centre would be classified as entirely separate from a 78 yr. old, man living in a rural village, on an experiential level the impact social prescribing has had on their lives would correlate.

Taken together, the findings point to the need for an evaluative logic capable of recognising prevention, stabilisation, emotional change, and the creation of conditions for engagement as legitimate forms of impact. For the participants in this study, whose lives were characterised by ongoing crisis, isolation, and depletion, the significance of social prescribing lay not in neatly measurable gains, but in averting further collapse and enabling the possibility of change. Recognising these forms of impact is not only a methodological concern, but a policy concern, as it has direct implications on how social prescribing is designed, assessed, and funded.

### 4.2 Relational labour, time, and the misrecognition of link worker practice

The findings also draw attention to a misalignment between how link worker practice operates in reality and how it is recognised within evaluation. Participants consistently positioned link workers not as coordinators of services, but as relational figures whose work involved sustained emotional and practical labour. This relational labour was not incidental but foundational. Engagement often depended on repeated contact, reassurance, accompaniment, and the sense that someone would continue to show up even when progress was slow or uncertain. Crucially, these practices unfolded over time, across weeks and months, rather than within the short cycles typically assumed by referral based or time limited interventions. These temporal characteristics sit uneasily within evaluation frameworks that prioritise rapid outcomes and fixed programme durations. Persistence, follow up, and availability are difficult to capture through standard metrics, particularly when success is defined in terms of course completion, or short-term wellbeing scores. Indeed, participants’ accounts suggest that some of the most consequential aspects of link worker practice occur before any measurable outcomes appear, and in many cases, it is the link worker that enables any engagement to happen at all.

Across the accounts analysed, the transformative potential described by participants appears to lie less in specific activities and more in the sustained experience of being accompanied. In many accounts, it was not the activity itself that was decisive, but the consistency of the link worker’s presence and the knowledge that someone would remain engaged even when progress was slow or interrupted. Relational labour therefore appears to function as a stabilising social anchor within lives often characterised by instability. In this sense, the relational work of the link worker holds uncertainty, distress, and fragmentation long enough for participation to become possible. This is particularly significant in contexts where other services are experienced as episodic, procedural, or contingent on compliance. The relational nature of link worker labour also challenges assumptions embedded within professional hierarchies and role definitions. Participants repeatedly contrasted link workers with clinicians, therapists, or statutory professionals, emphasising that what made the difference was not specialist expertise but the quality of human connection. Being listened to, believed, and treated without judgement were described as transformative precisely because such experiences had been absent elsewhere. Yet these qualities are rarely specified, or resourced within service models, which tend to frame link worker roles in terms of navigation or signposting rather than relational care.

This misrecognition has practical consequences. When relational labour is not adequately acknowledged, it risks being rendered expendable under conditions of constrained resources. Participants’ accounts of disrupted engagement following changes in link worker availability illustrate the fragility of progress when continuity is lost. Outcomes may stall or reverse not because social prescribing has failed, but because the relational conditions (namely the link worker) that made engagement possible have been overstretched and under-resourced. Therefore, recognising relational labour as a core component of social prescribing is not simply a matter of professional acknowledgement, but of methodological alignment. Without evaluative approaches capable of capturing the integral role link workers play, their contribution will continue to be underestimated. As such, evaluation frameworks have an important part to play in shaping how the link worker role is understood, valued, and sustained.

### 4.3 Implications for evidence-based public health policy

The findings of this research have important implications for how social prescribing is evaluated, commissioned, and justified within public health policy. Current approaches focus on short-term efficiency, positioned against a backdrop of constrained resources. However, the accounts presented here suggest that the frameworks used to assess social prescribing are poorly aligned with the realities of how engagement is enabled and sustained. From a commissioning perspective, this creates a very clear problem. Models that emphasise short delivery cycles, rapid outcomes, or demonstrable cost savings risk undervaluing the very forms of work that enable social prescribing to function effectively.

The findings also raise broader questions about how “evidence” is conceptualised. Participants’ accounts demonstrate that change is often slow, fragile, and non-linear, shaped by cumulative life histories rather than discrete interventions. Improvements may involve increased engagement with services, greater willingness to seek help, or the surfacing of previously unmet needs (developments that may even register negatively within conventional evaluation frameworks). Evaluation approaches that treat reduced service use or short-term wellbeing gains as primary indicators of success are therefore likely to misinterpret these trajectories and overlook the long-term preventive role social prescribing can play. Importantly, the findings suggest that qualitative evidence should not be positioned as supplementary or illustrative, but as structurally necessary within social prescribing evaluation. Narrative accounts reveal that trust building, persistence, and emotional safety are central to enabling engagement. However, these aspects remain largely invisible to quantitative measures.

Recent national research on the social prescribing workforce further underscores these concerns, highlighting link workers’ commitment to relational practice alongside persistent challenges such as the role being misunderstood, evaluative pressure to evidence impact, and limited recognition within primary care systems (21, 22). Read alongside the findings of this study, such evidence suggests that current evaluation and commissioning frameworks may be exerting pressure on precisely those relational and temporal dimensions of practice that participants identify as most valuable. The risk then is that social prescribing is expanded in form while being hollowed out in substance.

### 4.4 Strengths, limitations, and future directions

This study offers a detailed qualitative account of how social prescribing is experienced by those who engage with it. A key strength lies in the depth and consistency of participants’ accounts across a relatively large qualitative sample. Drawing on 40 in-depth interviews conducted in person, the analysis captures patterns of experience that extend beyond isolated cases, allowing for thematic development grounded in lived experience. The study also benefits from strong methodological coherence. The qualitative design is well suited to examining processes that unfold over extended periods and within complex social contexts, and the analytic approach enables attention to both individual narratives and recurring patterns across the dataset. By privileging participants’ own language and framing, the analysis avoids imposing externally defined success criteria. Instead, it reveals how the impact of social prescribing is understood by those that have directly engaged with it.

Several limitations should be acknowledged. The findings are based on a single geographic area and reflect the experiences of participants who were able and willing to take part in interviews. While this limits statistical generalisability, the aim was not to produce representative prevalence estimates, but to generate analytic insight into mechanisms, processes, and forms of value that are not captured in existing evaluation approaches. Transferability therefore lies in conceptual rather than demographic relevance. However, while grounded in a specific geographic context, the dynamics identified here (cumulative adversity, relational engagement, and evaluative misalignment) are not unique to this setting.

Recruitment via link workers introduces the possibility of selection bias. It is plausible that individuals who had engaged positively with social prescribing, or who maintained relationships with their link worker, may have been more likely to accept an invitation to participate. Similarly, those who disengaged early or held strongly negative views may be underrepresented. Although three participants had disengaged from services, and a small number described negative or disrupted experiences, the sample may overrepresent those who sustained engagement. In addition, trust in link workers is likely to have informed participant’s willingness to consider participation. However, this trust did not automatically transfer to the researchers. Several participants expressed anxiety prior to interview, particularly where initial contact occurred by email alone. As participants were not actively receiving structured support at the time of interview, the risk of social desirability bias linked to ongoing care relationships was reduced. Nonetheless, the possibility that positive experiences were more likely to be shared cannot be entirely discounted.

The study also relies on retrospective accounts and does not include observational data or prospective longitudinal follow up. Future research could extend this work by combining qualitative life history approaches with longer-term follow up to examine how engagement with social prescribing evolves over time. There is also scope for research that brings together participant narratives, link worker perspectives, and commissioning data to explore how evaluation frameworks actively shape practice.

## 5 Conclusion

Taken together, the findings of this research point to the need for a recalibration of how social prescribing is evaluated. Crucially, this requires not only different methods, but different evaluative questions designed to capture stabilisation, safety, trust, persistence, and the prevention of further deterioration. This does not require abandoning quantitative measurement, but expanding what is considered legitimate and possible to measure. Such a shift would mark a move away from narrow outcome metrics and towards pluralistic evaluative approaches capable of accommodating relational labour, emotional depth, and fragile or preventative forms of progress. This would include the development of new instruments and analytic approaches capable of capturing trajectories, starting points, and changes that are currently invisible in standardised wellbeing scales. Without such recalibration, commissioning and evaluation systems are likely to continue rewarding what is most easily counted, rather than what is most effective, thereby reinforcing the gap between lived experience, workforce practice, and the evidence base used to justify investment and policy. Put simply, aligning evaluation more closely with lived experience is essential if social prescribing is to be assessed, commissioned, and sustained in ways that reflect how it actually works.

## Data Availability

The datasets presented in this article are not readily available because of ethical and confidentiality considerations. The data comprises of in-depth qualitative interviews containing sensitive personal information relating to mental health, trauma, and lived experience within a geographically bounded population. In accordance with the terms of ethical approval and participant consent, the dataset cannot be shared beyond the research team, as doing so could risk participant identification or distress. Requests to access the datasets should be directed to RD, rdean@lincoln.ac.uk.
